# A computational biomechanics study to investigate the effect of myoelectric stimulation on peroneal muscles in preventing inversion-type ankle ligamentous sprain injury

**DOI:** 10.1186/1757-1146-7-S1-A29

**Published:** 2014-04-08

**Authors:** Sophia CW Ha, Jianxin Chen, Daniel TP Fong, KM Chan

**Affiliations:** 1 Department of Orthopaedics and Traumatology, Prince of Wales Hospital, Faculty of Medicine, The Chinese University of Hong Kong, Hong Kong; 2 School of Sport, Exercise and Health Sciences, Loughborough University, Leicestershire LE11 3TU, UK

## Introduction

The aim of this study was to develop a three-dimensional (3D) computational model to justify the effect of myoelectric stimulation in preventing ankle inversion ligamentous sprain injury.

## Methods

The subject who sustained a grade 1 anterior talofibular ligamentous sprain injury on his right ankle during our previous case report [1] was invited to participate in this project. There were three steps: 1) Computational model development: CT scan was performed from mid-femur to the foot segments. The CT images were separated and meshed as individual solid bodies in MIMICS. These individual bones were computationally separated and meshed in STL files. These files will be remeshed in MIMICS to smooth each bone in order to reduce the file size. The 3D computational lower limb model was then imported into SolidWorks for applying ligamentous restraints, prescribing force, motion constraints, prescribing muscle forces, and simulating the ankle dynamics. 2) Model validation: This model will be validated against two cadaveric studies. 3) Model simulation: A systematic series of simulations will be conducted to deliver the myoelectric stimulation when different ankle inversion velocity threshold is achieved, at different delay time, and at different stimulation.

## Results

The model is validated. Stimulation with a delay time of 25 milliseconds could successfully prevent the ankle inversion sprain when the lower threshold of 300 or 400 degrees per second was identified.

## Conclusion

This study is indispensable and crucial for evaluating the actual effect of myoelectric stimulation on peroneal muscles in preventing ankle inversion sprain injury. Meanwhile, this study would also contribute to the research on the intelligent anti-sprain system, which in turn would boost sports participation with more effective protection for the general public.
